# Lipid from Infective *L*. *donovani* Regulates Acute Myeloid Cell Growth via Mitochondria Dependent MAPK Pathway

**DOI:** 10.1371/journal.pone.0120509

**Published:** 2015-03-09

**Authors:** Nabanita Chatterjee, Subhadip Das, Dipayan Bose, Somenath Banerjee, Tarun Jha, Krishna Das Saha

**Affiliations:** 1 Cancer Biology & Inflammatory Disorder Division, CSIR-Indian Institute of Chemical Biology, 4 Raja S.C. Mullick Road, Kolkata-700032, West Bengal, India; 2 Division of Medicinal and Pharmaceutical Chemistry, Department of Pharmaceutical Technology, P. O. Box 17020, Jadavpur University, Kolkata 700032, India; CDFD, INDIA

## Abstract

The microbial source, which includes live, attenuated, or genetically modified microbes or their cellular component(s) or metabolites, has gained increasing significance for therapeutic intervention against several pathophysiological conditions of disease including leukemia, which remains an incurable disease till now despite recent advances in the medical sciences. We therefore took up the present study to explore if the leishmanial lipid (pLLD) isolated from *L*. *donovani* can play an anti-neoplastic role in acute myeloid leukemia cells by regulating cellular growth. Indeed pLLD significantly inhibited cell proliferation of four AML cell lines (HL-60, MOLT-4, U937, and K562). Scanning electron microscopy and DNA fragmentation analysis revealed that it significantly induced apoptosis of U937 cells through morphological alteration. Occurrence of apoptosis was checked by using Annexin exposure and this established that the cell cycle was arrested at G0/G1 phase in time-dependent manner. pLLD increased the intracellular ROS with alteration of mitochondrial membrane potential, as detected using DCFDA. It also regulated the expression of apoptosis-related proteins like Bax, Bcl2, Bad and t-Bid besides causing cleavage of PARP as determined by western blot analysis. Treatment of U937 cells with pLLD induced the activation of extracellular signal-regulated kinase (ERK)1/2, c-Jun N-terminal kinase (JNK)1/2, p38, and caspases 9/3. The results suggest that pLLD induces apoptosis in acute myeloid leukemia cells possibly via increasing intracellular ROS and regulating the MAPK pathway.

## Introduction

Leukemia is a heterogeneous cluster of neoplasm arising from the malignant transformation of haematopoietic cells [1–4]. Specifically, acute myeloid leukemia (AML), a myeloid line of blood cells characterised by the failure to differentiate and by over proliferation in the stem cell compartment, leads to accumulation of non-functional myeloblast cells [5, 6]. Treatment by chemotherapeutic exposure may even increase the chances of subsequently developing AML; the risk is highest about three to five years after chemotherapy [7]. Conventional therapy of AML is a combination of debilitating radiation exposure and bone marrow transplantation that induces complete remission in only 50% of patients with recovery rate of 30%. Moreover, these chemotherapeutic agents can also affect the health of normal cells causing unpleasant side effects such as anemia, bleeding, and infection [8]. Thus, these strategies have not proven to be altogether satisfactory.

New approaches for leukemia treatment are therefore essential, and this may be brought about by promoting differentiation or triggering apoptotic death in leukemic cells. Apoptosis is a biochemical process with structural alteration and can be induced by chemotherapeutic agents mostly via mitochondrial apoptotic pathways [9, 10]. The release of mitochondrial apoptotic proteins affects cellular ionic imbalance. This initiates cytochrome c release and leads to activation of caspase-9, which in turn triggers the signaling cascade causing caspase-dependent DNA fragmentation. Thus, the involvement of several pro-apoptotic (e.g., Bax, Bak, Bad) or anti-apoptotic (e.g., Bcl-2, Bcl-xL) molecules regulate the mitochondrial membrane permeability in apoptosis under the stimulation of reactive oxygen species [11]. Bcl-2 family members act as important prognostic factors in AML; the inhibition of Bcl-2 expression induces apoptosis and sensitizes AML cells to chemotherapy via regulation of mitogen-activated protein kinase pathway, a key integration point in the signaling cascade regulated by growth factor receptors. Constitutive regulation of the MAPK signaling and related cascade may drive the oncogenic transformation of normal blast cells and is commonly detected in myeloid leukemia resulting from a variety of genetic alterations recognized to be responsive against AML [12].

Natural products that may even come from microbial sources have gained increasing importance for potential use in intervention against malignant neoplastic diseases. One such example involves the use of live, attenuated bacteria or their metabolites and cellular components to improve the therapeutic efficacy. Often anerobic bacteria mediated therapy or use of several bacterial products like LPS or ceramide(s) potentially induce apoptosis in malignant cells [13, 14]. Interestingly, a lipid molecule isolated from a fungus has shown its therapeutic efficacy in multiple sclerosis and neoplasia [15]. Previously, we reported that the lipid from the avirulent strain of *L*. *donovani* plays a potential role in arthritis patients to control the inflammatory response [16]. Very recently, we have also explored the bio-activity of virulent leishmanial lipid (pLLD) in protection against sepsis associated inflammatory injury [17]. This interesting outcome encouraged us to reveal the therapeutic efficacy of microbes and their metabolites, in particular the leishmanial lipid (pLLD). Herein we disclose a new therapeutic role of pLLD to induce apoptosis in AML cells following the mitochondrial apoptotic pathway of activation of caspases and MAPK signaling proteins.

## Materials and Methods

### Material

MTT and DMSO were purchased from Sigma Chemical Co. (St. Louis, MO, USA); p-NBT-BCIP systems were from Amresco (Solon, OH, USA); RPMI 1640, FBS (Fetal Bovine Serum), and penicillin—streptomycin—neomycin were from Gibco-BRL (Grand Island, NY, USA); tissue-culture plasticware was from Nunc (Roskilde, Denmark); Bradford protein assay reagent was from Fermentas (Pittsburgh, PA, USA); DAPI (4',6-diamidino-2-phenylindole dihydrochloride), acridine orange (AO), and ethidium bromide (EtBr) were from Invitrogen (Carlsbad, CA, USA); rabbit and goat anti-BAX, BAD, BCL-2, p38, p-P38, ERK, p-ERK, JNK polyclonal and secondary antibodies in alkaline phosphatase, and FITC and PE-conjugated were from Santa Cruz Biotechnology (Santa Cruz, CA, USA).

### *L*. *donovani* cell culture and isolation of lipid

*L*. *donovani* strain AG83 (MHOM/IN/1983/AG83) used for the present experiments was obtained originally from Indian kala-azar patients and maintained in golden hamsters [18]. Promastigotes obtained after transforming amastigotes from infected hamster spleen were maintained at 22°C in M199 (Invitrogen), supplemented with antibiotics and 10% FBS. The Bligh and Dyer method [19] of lipid extraction was used to isolate the total lipid from *Leishmania* cells (1×10^10^). The total lipid, obtained from the lower organic phase after evaporation to dryness at 40°C, was then stored at 4°C in vacuum desiccators until used.

### Thin layer chromatography

pLLD was dissolved in 2:1 chloroform:methanol. TLC was performed in chloroform:methanol:water (30:60:10) and lipid spots were visualized using iodine spray [17].

### Cell culture

Human leukemic monocyte lymphoma cells (U937), human chronic myelogenous leukemia cells (K562), human promyelocytic leukemia cells (HL-60), and human acute lymphoblastic leukemia cells (MOLT-4) of American Type Culture Collection [ATCC] were used. The cells were cultured in RPMI or DMEM supplemented with 10% FBS and 1% antibiotic PSN at 37°C in a humidified atmosphere under 5% CO_2_. After 75–80% confluency, cells were harvested at desired density to allow them to re-equilibrate a day before the start of experiment.

### Cell viability assay

MTT assay was done to evaluate cell viability [20]. The cells were seeded in 96-well plates and treated with or without different concentrations of pLLD for 0–36 h. Four hours after the addition of MTT, cells were lysed, formazan was solubilized with acidic isopropanol, and the absorbance of the solution was measured at 595 nm using an ELISA reader as also by flow cytometric analysis using propidium iodide.

### Assessment of cell morphology

Cells (3×10^4^/well) were grown in 30 mm culture plates after treatment with pLLD. Morphological changes were observed with an inverted phase contrast microscope. To detect nuclear damage or chromatin condensation, cells were treated with 10 μg/ml of DAPI. The conventional acridine orange/ethidium bromide (AO/EtBr) staining procedure followed by observation under fluorescence microscope (OLYMPUS IX70, Olympus Optical Co. Ltd) was used to differentiate the live, apoptotic and necrotic cells and images were acquired with excitation and emission wavelengths of 488 and 550 nm respectively [21].

### Apoptosis assay using Annexin-V

Apoptosis was assayed by using an Annexin-V FITC apoptosis detection kit (Calbiochem, CA, USA). Briefly, cells were treated with pLLD, and then washed and stained with PI and Annexin-V-FITC in accordance with the manufacturer's instructions. The percentages of live, apoptotic and necrotic cells were determined by flow cytometric method using the equipment LSR Fortess of Beckton Dickinson, San Jose, CA, USA [22]. Data from 10^6^ cells were analyzed for each sample.

### Cell cycle analysis

Upon treatment, cells were collected and fixed in 70% ethanol for 24 h at 4°C. These were centrifuged (1500 g) and the cell pellet was resuspended in PBS (400 μl), containing RNaseA (10 mg/ml, 50 μl) and PI (2 mg/ml, 10 μl). The mixture was incubated in the dark at 37°C for 30 min and analysed by flow cytometry [21].

### Reactive oxygen species (ROS) assay

ROS were detected using the cell-permeable fluorescence probe 2,7-dichlorofluorescein diacetate or H2DCFDA (Sigma-Aldrich, USA), a non-fluorescent compound, which is converted into the highly fluorescent dichlorofluorescein (DCF) by cellular peroxides. Briefly, cells were exposed to various agents for the indicated times and then loaded with H2DCFDA (20 mM). Following incubation at 37°C for 30 min, cells were washed with PBS and fluorescence assayed by flow cytometric method at excitation wavelength of 488 nm and emission wavelength of 530 nm.

### Mitochondrial membrane potential (MMP/Δψm) measurement

Cells were treated with the voltage-sensitive lipophilic cationic fluorescent probe 5,5',6,6'-tetrachloro-1,1',3,3'-tetraethyl-benzimidazolylcarbocyanine iodide (JC-1) to measure MMP. JC-1 monomers fluorescence red in stable mitochondria but combine to form green fluorescent dimers upon exclusion. Briefly, cells were collected, washed with cold PBS, incubated with JC-1 (5 mg/ml) for 15 min, and analyzed by flow cytometric method.

### Confocal microscopy

During apoptosis induction in U937 cells, released cytochrome C and AP-1 were measured by immunocytochemical analysis. Cells cultured on chambered plastic slides were fixed with ethanol for 30 min at 4°C and the detergent was extracted with 3% Triton X-100 for 10 min at room temperature. After washing with PBS and blocking with 3% bovine serum albumin (BSA) for 30 min, samples were incubated overnight with a primary antibody at 4°C. Excess primary antibody was removed by washing with PBS and samples were incubated with FITC-conjugated secondary antibody for 2 h at room temperature; subsequently mitochondria were stained with Mito Red. After washing with PBS, slides were mounted using DAPI to visualize the nuclei. Specimens were covered with cover slips and evaluated under an Andor spinning Disc laser scanning confocal microscope [23].

### Scanning electron microscopy (SEM) analysis for morphological study

Cells were incubated with or without pLLD, washed with PBS, and then pelleted by low-speed centrifugation. The pelleted cells were prefixed with 2.5% glutaraldehyde in PBS for 2 h, rinsed with PBS, and post fixed with 1% osmium tetroxide in PBS for 2 h. The samples were dehydrated by a series of ethanol rinses followed by critical-point drying using an HCP-2 apparatus (Hitachi, Tokyo, Japan) employing CO_2_ as the transitional fluid. The specimens mounted on stubs were coated with platinum, examined with a scanning electron microscope (S-4500; Hitachi), and photographed [24].

### Western blot analysis

The cell lysates were separated by 10% SDS-PAGE and transferred to PVDF membranes (Millipore, Bedford, MA) using standard electroblotting procedures. Membranes were then blocked and immunolabeled overnight at 4°C with primary antibodies. Alkaline phosphatase conjugated secondary antibodies and NBT-BCIP were used as chromogenic substrates [23].

### Statistical analysis

Results were expressed as mean ± SEM. Statistical analyses were performed with ANOVA, followed by Dunnett’s test. P < 0.05 was considered significant.

## Results

### Effect of leishmanial lipid on the growth of leukemic cell lines

**Fig 1:**
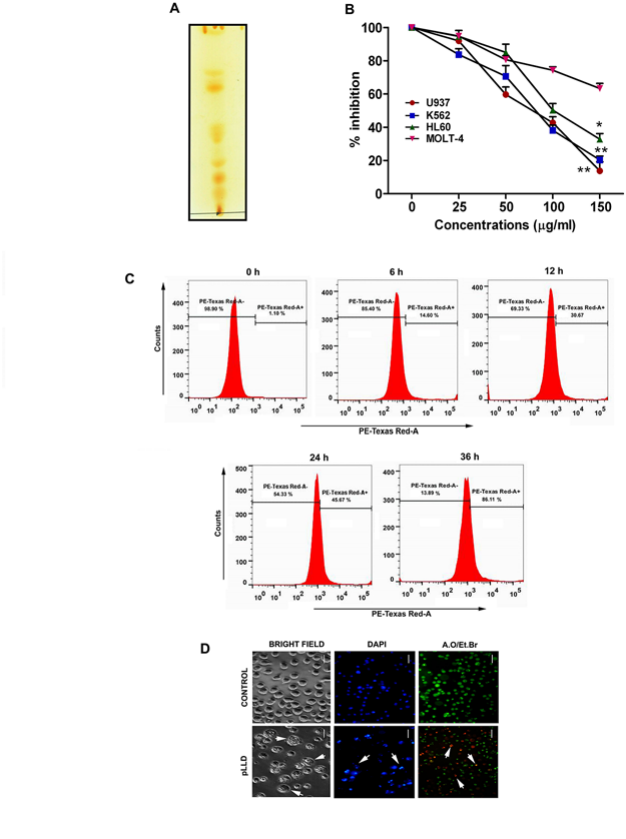
TLC profile of pLLD (A). Growth inhibitory effect of pLLD on different cancer cells including U937, K562, HL-60 and MOLT-4 at different concentrations (0, 25, 50, 150, 200 μg/ml) at 24 h; viability was measured by MTT assay (B). The data are represented as mean ± SEM from triplicate independent experiments. (*P>0.05; ** P>0.01) Viability of U937 cells was measured in time dependent manner by flow cytometry after treatment with pLLD at the concentration 150 μg/ml (C). Morphological and nuclear changes observed in U937 cells after treatment of 150 μg/ml pLLD for 24 h (D). Morphological changes were seen under light microscope, and nuclear changes (Right to left) under fluorescence microscope after DAPI and A.O./EtBr staining respectively.

The TLC profile of pLLD used in our study showed six to seven spots of lipids in the TLC plate (Fig. 1A) upon iodine staining; this product was used for further studies. To determine the cytotoxicity and the effect on cell proliferation, four AML cell lines (U937, K562, HL-60 and MOLT-4) were treated with different concentrations (0–200 μg/ml) of pLLD (Fig. 1B). Treatment with pLLD significantly reduced the cell viability of the four AML cell lines in a time-dependent manner as compared with the percent growth inhibition in normal blood cells and other cancerous cells as shown in S1 Fig. But the highest potency was observed in U937 cells. Treatment with 150 μg/ml pLLD followed by observation under light microscope using the nuclear staining dye DAPI showed characteristic apoptotic changes like cell rounding and cell shrinkage at 24 h, while AO/Et.Br showed the presence of condensed and fragmented nuclei unlike untreated control in U937 cells. DAPI staining showed bluish intact nuclei in control and bright fragmented nuclei in the treated cells. AO/Et.Br treatment showed green intact nuclei in control cells, but greenish yellow, yellowish red and reddish fragmented nuclei in U937 treated cells (Fig. 1D). Thus the microscopic study indicated that pLLD induced death in U937 cells may involve apoptosis.

### pLLD induces apoptosis and cell cycle arrest in U937 cells

**Fig 2:**
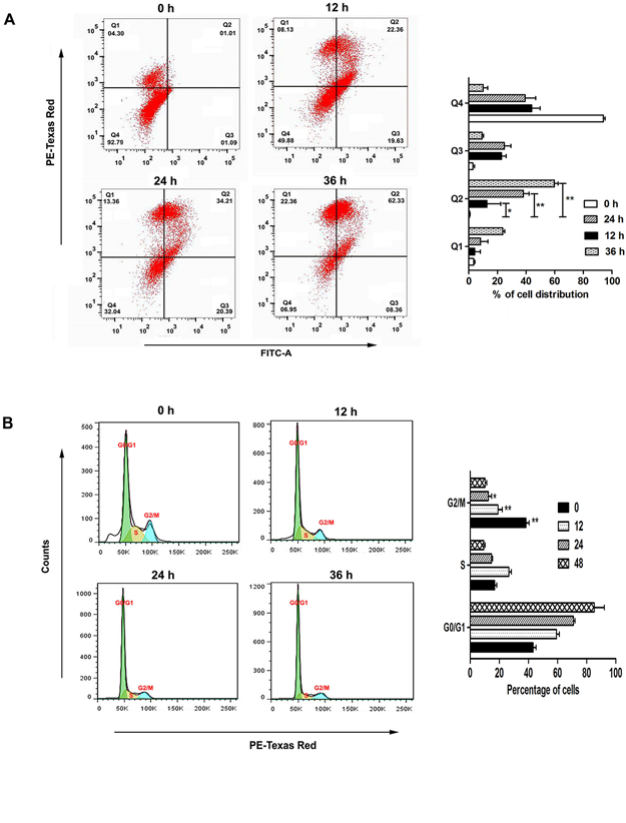
Analysis of apoptosis and cell cycle arrest in U937 cells by flow cytometry. Cells were treated with 150 μg/ml of pLLD and binding of Annexin-V/FITC to phosphatidyl serine was measured by flow cytometry to determine the percentages of apoptotic cells in time dependent manner (A). Study of cell cycle arrest in U937 cells was carried out by propidium iodide staining. Percentage of G0/G1 cell population increases after treatment of 150 μg/ml of pLLD in time dependent manner (B). The data are represented as mean ± SEM from triplicate independent experiments (*P>0.05; ** P>0.01).

At the initial phase of apoptosis, phosphatidyl serine is exposed from the inner membrane to the outer membrane of the cell and can bind with Annexin V. This can be detected through flow cytometric analysis of phosphatidyl serine bound to Annexin V tagged with FITC. The results of flow cytometric analysis showed a higher number of Annexin V positive cells at 24 h in U937 cells treated with 150 μg/ml of pLLD than in the control (Fig. 2A). Cell cycle analysis by flow cytometry also demonstrated a time-dependent increased accumulation of cell population in sub-G1 phase (Fig. 2B). The cytotoxicity induced by pLLD in U937 cells may be responsible for the increased percentage of apoptosis detected after 24 h.

### Effect of pLLD on blebbing in U937 cells

**Fig 3:**
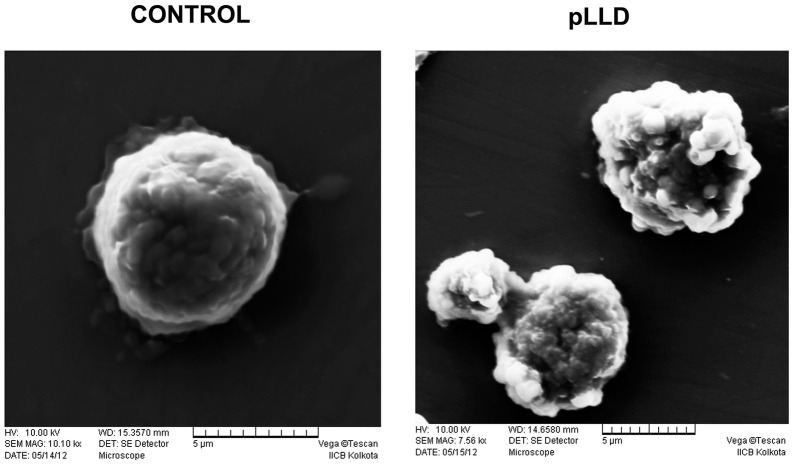
Ultra-structural changes of U937 cells incubated for 24 h with or without 150 μg/ml of pLLD by scanning electron microscopy.

Blebbing in cellular surface is a dispensable event of apoptosis. Cell shape alterations and blebbing are strikingly rapid phenomena showed by the irregular round shape of the cells with many pseudopodia-like protrusions as found during apoptosis. The smooth round shape is replaced with protruding spherically shaped blebs of apoptotic cells which can be noted by scanning electron microscopy. The presence and distribution of blebs in the U937 cells at 24 h upon treatment of 150 μg/ml of pLLD was indeed observed (Fig. 3).

### pLLD induced activation of caspases-3 and -9 and its associate proteins in U937 cells

**Fig 4:**
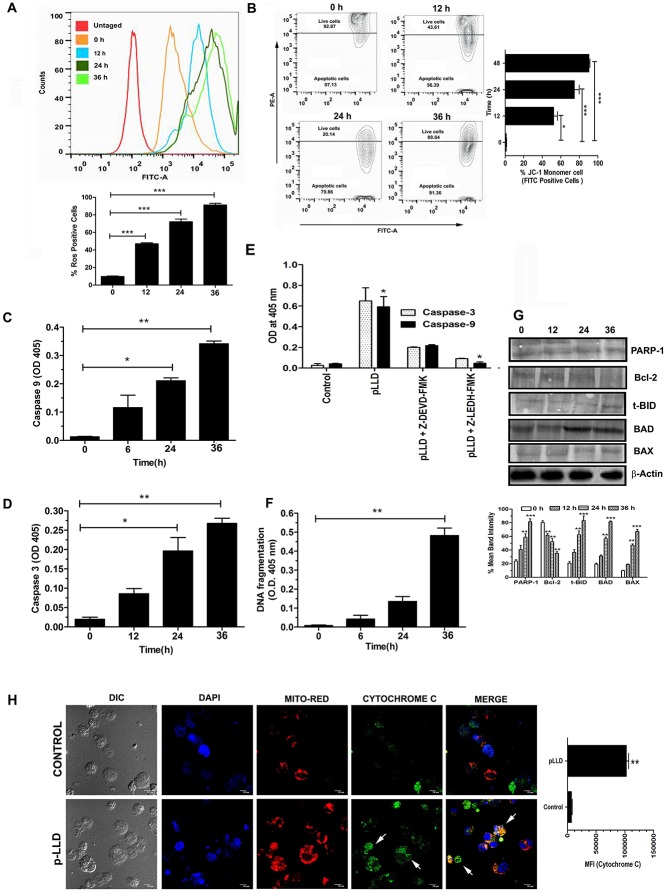
ROS generation in U937 cells upon treatment with pLLD (150 μg/ml). Flow cytometric analysis of ROS by DCFDA showed the degrees of scavenging effect in time dependent manner (A). Evaluation of mitochondrial membrane potential using JC-1 showed time dependent changes (B). Assessment of activation of caspases 9 and 3 with inhibitors and of DNA fragmentation by pLLD was done in a time dependent analysis by ELISA (C, D, E and F, respectively). Expression of pro- and anti-apoptotic molecules was assessed by western blot (G). Analysis of cytochrome C activation upon pLLD treatment was performed by confocal microscopy (H). The data are reported as the mean ± SEM of triplicate experiments (*P>0.05; ** P>0.01; *** P>0.001).

To confirm the involvement of caspase activation in pLLD-induced apoptosis, it was necessary to detect the activation of caspases-3 and -9 with cleavage of PARP, as DNA fragmentation is one of the important events of cellular apoptosis. Using the ELISA based cell death detection kit assay, we found that treatment of pLLD increased the level of fragmented DNA in U937 cells (Fig. 4F). DNA fragmentation pattern expectedly increased in due time; high level of DNA fragmentation and the occurrence of apoptosis process were observed with 150 μg/ml of pLLD at 24 h. Fig. 4C and D show that the exposure of U937 cells to pLLD (150 μg/ml) caused gradual increase of the cleaved fragments of caspases-9 and -3 in time dependent manner. Cleaved PARP level was also significantly increased in pLLD treated U937 cells at 24 h (Fig. 4G). Further, cells pre-incubated with Z-LEHD-FMK and Z-DEVD-FMK exhibited lower level of expression of caspases 3 and 9 as compared to cells pretreated with either or none of these inhibitors (Fig. 4E and S3 Fig.). pLLD caused time-dependent decrease in the expression of Bcl-2; in addition, the expression levels of truncated Bid, Bax and Bad were increased (Fig. 4G).

### Involvement of mitochondrial pathway in pLLD induced apoptosis

As U937 cell apoptosis was triggered by pLLD via activation of caspase-9, it indicated the involvement of mitochondrial pathway. This was confirmed by measuring the status of cytosolic cytochrome C and the loss of mitochondrial membrane potential. Cytosolic cytochrome C level was increased in pLLD treated cells as found by confocal microscopy at 24 h (Fig. 4H). Down-regulation of the anti-apoptotic Bcl-2 protein and binding of pro-apoptotic Bax protein to the mitochondrial membrane trigger the release of cytochrome C from mitochondria to cytosol and generation of ROS. Flow cytometric analysis for determining the mitochondrial membrane potential in U937 cells revealed that 7% of the cell population exhibited fluorescence at the FITC channel as against 56%, 79% and 91% of the cell population receiving 150 μg/ml pLLD in respective time (Fig. 4B); this indicated a higher level of cells having a healthy ΔΨm in the control population. Time dependent of depletion of intracellular GSH correlates with that of detection of intracellular ROS. S2 Fig. suggests that enhanced depletion of intracellular GSH may be responsible for production of enhanced ROS in the presence of pLLD in leukemic cells.

Generation of ROS is a key factor in apoptotic cell death. Indeed maximum ROS generation at due time was exhibited with pLLD treatment at 150 μg/ml, as found by flow cytometric analysis (Fig. 4A). The study demonstrates that pLLD induced apoptosis in U937 cells may proceed via the ROS mediated pathway.

### Regulation of MAPK signaling with pLLD induced apoptosis in U937 cells

**Fig 5:**
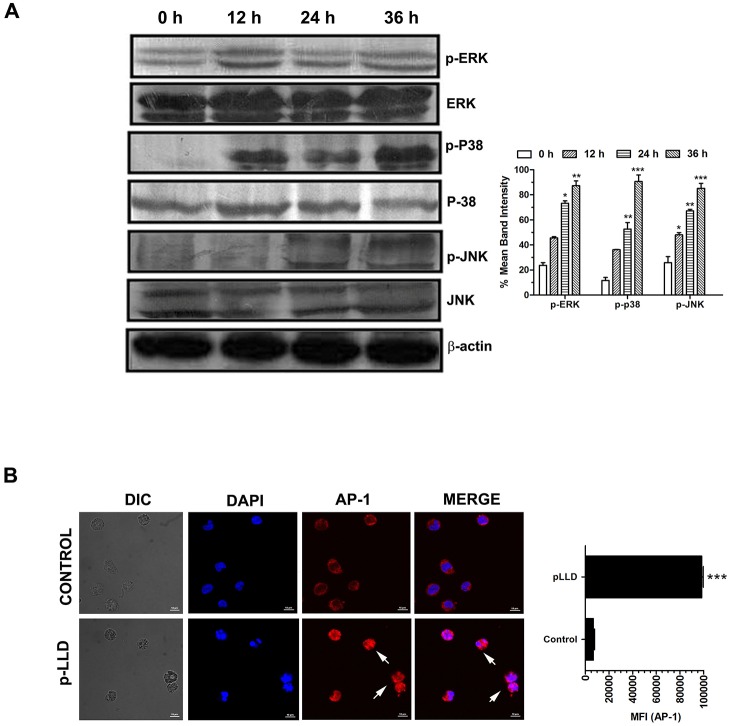
The effect of pLLD on MAPK pathway in U937 cells. Representative western blot results show phospho-ERK1/2/ERK1/2, phospho-JNK/JNK and phospho-p38/p38 expressions in U937 cells after incubation with pLLD (A). AP-1 expression in U937 cells 24 h after treatment of pLLD, evaluated by confocal microscopy (B) (*P>0.05; ** P>0.01; *** P>0.001).

Of the several signaling pathways that have been implicated in the action of chemotherapeutic drugs in the regulation of apoptosis, MAPK signaling pathway is an important one. For investigating the underlying molecular mechanisms, we therefore employed western blot analysis to determine whether MAPKs were activated in pLLD-treated U937 cells. The results showed that ERK1/2, JNK1/2 and p38 MAPK levels were altered. The treatment of U937 cells with pLLD (150 μg/ml) also resulted in a time-dependent increase in the phosphorylation of ERK1/2, JNK1/2 and p38 MAPK (Fig. 5A and S3 Fig.). Furthermore, immuno cytochemical analysis of treated cells showed transcriptional changes in AP-1 expression; the transcriptional activity was transiently enhanced at 24 h (Fig. 5B). Thus, these apoptotic changes may have proceeded via dependence of MAPK signaling on involvement of activator protein-1.

## Discussion

Several microbes including eukaryotic and prokaryotic organisms and their cellular component(s) may possess a range of bioactivities against pathological circumstances of our body [13, 25]. For example, the sophorolipid of *Wickerhamiella domercqiae* shows potential activity against human pancreatic cancer cells [26]. Azurin, a protein isolated from the pathogenic bacteria *Pseudomonas aeruginosa*, induces apoptosis by interfering with signaling molecules and regulating the cancer cell growth [27]. The microbial metabolites myriocin of *Isaria sinclairii* and 6-MFA of *Aspergillus ochraceus* (as also of one marine sponge *Hippospongia communis*) afford protection against hepatic injury [28]. Interestingly, in addition to the potent leishmanicidal activity, *L*. *amazonensis* and its components also inhibited the expression of nitric oxide synthase (iNOS) in stimulated macrophage cells [29, 30]. A previous study has shown that the lipid fraction of *L*. *donovani* suppresses host immune responses [16]. Earlier, we have reported the bio-activity of the leishmanial lipid in protecting against sepsis associated inflammatory injury [17]. Lipids are ubiquitous components of eukaryotic cell membranes that play diverse physiological roles in mammalian cells such as in cell differentiation, proliferation and/or receptor mediated endocytosis. Importantly, they have been shown to constitute a messenger system that plays a crucial role in the induction of apoptosis via activation of cellular signal transduction systems [31]. These interesting findings inspired us to find out a novel entity from microbial source by exploring the biological potency of the leishmanial lipid for the regression of cancer cell growth in AML.

AML is a progressive non-solid tumorigenesis event with high mortality rates, for which novel strategies are needed to improve the current scenario [7]. Our studies demonstrated that pLLD concentration-dependently inhibited cell proliferation in four human leukemic cell lines, viz. U937, HL-60, K562 and MOLT-4. Cell proliferation is governed by Annexin exposure and cell cycle, apoptosis such as significant increases in chromatin condensation, that is a complex and stepwise process, and uncontrolled cell proliferation is a hallmark of cancer [32]. We have performed the cytotoxicity assay to observe that 150 μg/ml of pLLD causes minimal viability in U937 cells at 24 h. Apoptotic like morphological and nuclear changes were also effected in pLLD treated U937 cells in time dependent manner. This was supported by the enhancement of phosphatidyl serine externalization which belongs to apoptotic events and increases apoptotic DNA cleavage with G1/G0 phase arrest. Using scanning electron microscopy, we found similar apoptotic like alterations including blebbing and swelling in treated U937 cells.

Apoptosis is mediated by proteolytic enzymes called caspases, which trigger cell death by cleaving specific proteins in the cytoplasm and nucleus. The activation process is initiated by extracellular or intracellular death signals which cause intracellular adaptor molecules to aggregate, generate ROS, and activate pro-caspases and then caspases [33]. Caspases 9 and 3 are believed to play crucial roles in mediating mitochondrion-mediated apoptosis pathway [34]. The present results suggest that pLLD might have acted through the initiator caspase-9 and then through the executioner caspase-3 to increase the cleavage form of PARP to induce AML cell apoptosis. Moreover, the inhibition of caspases 9 and 3 with Z-LEHD-FMK and Z-DEVD-FMK generally decreased the growth inhibitory potential of pLLD. Intracellular GSH depletion is an early hallmark in the progression of cell death in response to different apoptotic stimuli. As GSH is one of the prime antioxidant within the cells, that GSH regulates apoptosis by preventing the accumulation of ROS. Here, we represented that pLLD radically enhanced the depletion of intracellular GSH from leukemic cells directing to generation of ROS. The study indicates that pLLD initiated apoptosis via the mitochondrial pathway.

During mitochondria dysfunction, the mitochondrial permeability potential (MMP) alters and cytochrome c is released from mitochondria to cytosol [35]. The opening of MMP is controlled by the Bcl-2 family members. Bcl-2 is present in the outer mitochondrial membrane, where it functions to suppress apoptosis via blocking cytochrome c release and binding to Apaf-1. In the presence of excess Bax, Bcl-2 is displaced from Apaf-1, which may promote apoptosis [36, 37]. In our study, a decrease in the ratio of Bcl-2/Bax protein occurred in the AML cell line U937 after treatment with pLLD, which caused MMP loss and cyt c leak out.

Based on the ability of the signaling cascade to lower the apoptotic threshold, regulation of the MAPK module has been proposed as a novel approach to treat leukemia [38] as it increases the sensitivity to various apoptotic stimuli, such as DNA-damaging agents, with alteration of Bcl-2 anti-apoptotic activity at a post-translational level for cellular growth and survival [39]. Several studies show that the ERK1/2 pathway possesses anti-apoptotic functions [40, 41], primarily due to the increased activation of Bcl-2 and Bcl-XL. On the other hand, JNKs are indispensable for apoptosis and appear to ensure pro-apoptotic signaling by phosphorylating Bam and Bmf, activating Bax to initiate apoptosis, and phosphorylating Bcl-2 both in vitro and in intact cells [42]. The signaling of p38 MAPK causes rapid inactivation of the ERK1/2 pathway and negatively regulates JNK activity. The phosphorylated p38 can also activate a wide range of substrates that are generated by these phosphorylation events [39]. Our previous report has shown that MAPKs can be induced via pLLD mediated anti-inflammatory responses. Thus, the evidence presented here suggests that pLLD up-regulates phospho-ERK, phospho-JNK, and phospho-p38, and decreases the Bcl-2/Bax ratio in leukemic cells. Thus, pLLD may induce apoptosis of the acute myeloid leukemia cells via increase of intracellular ROS, activation of the p38 pathways, and inhibition of expression of the downstream mediator Bcl-2 [43].

AP-1, a transcriptional factor consisting of the dimers of members of the Jun families of proteins, appears to be responsible for apoptosis in leukemic cells. MAPKs share several upstream regulators and are involved in the regulation of transcription factors including AP-1 to induce biological changes, including cell proliferation, apoptosis, and differentiation. Thus, treatment of U937 cells with pLLD caused transient activation of AP-1, that is often regulated by Bcl2 [44].

In conclusion, this study indicates that pathogenic leishmanial lipid (pLLD) could have potential growth regulatory role in acute myeloid leukemia cells via inducing apoptosis. It stimulates the activation of caspases 3 and 9 with generation of ROS and alteration of mitochondrial membrane potential. This eventually results in the cleavage of PARP in U937 cells. It also moderately activates the pro and anti apoptotic proteins in leukemic cells. Interestingly, it activates the phosphorylation of JNK1/2 and p38 MAPK, and inhibits the expressions of Bcl-2 and Bid. Thus pLLD may prove to be a useful candidate for chemotherapy against AML.

## Supporting Information

S1 Fig Growth inhibitory effect of pLLD on different cancer cells including (A) A375, MCF-7, A549, PC and (B) normal blood peripheral mononuclear cells at different concentrations (0, 25, 50, 150, 200 μg/ml) at 24 h; viability was measured by MTT assay. Data represent mean ± SEM of three experiments (** p<0.01).
(DOC)

S2 Fig Effect of pLLD potentiates on intracellular GSH in U937 cells. U937 cells were treated as indicated for measurement of intracellular GSH as described in Materials and Methods. Data represent mean ± SEM of three experiments. ** p<0.01 and *** p<0.001.
(DOC)

S3 Fig (A) Assessment of caspase- 9 and 3 activation with inhibitors Z-DEVD-FMK, Z-LEDH-FMK by pLLD was done in a time dependent manner by western blot analysis. The data are reported as the mean ± SEM of triplicate experiments (*P>0.05; ** P>0.01). (B) Assessment of MAPK pathway with inhibitors PD098059, SB203580 and SP600125 by pLLD was done in a time dependent manner by western blot analysis. The data are reported as the mean ± SEM of triplicate experiments (*P>0.05; ** P>0.01; *** P>0.001).
(DOC)
