# Supplementary material for: Lipid from Infective L. donovani Regulates Acute Myeloid Cell Growth via Mitochondria Dependent MAPK Pathway
Source: PLoS One. 2015 Mar 9;10(3):e0120509. doi: 10.1371/journal.pone.0120509 (PMC4353703; doi:10.1371/journal.pone.0120509)
Supplement: S1 Fig — Data represent mean ± SEM of three experiments (** p<0.01). (DOC) [file pone.0120509.s001.doc]

**Supplementary Information 1**

**Lipid from infective *L. donovani* regulates acute myeloid cell growth via mitochondria dependent MAPK pathway**

Nabanita Chatterjee,^a^ Subhadip Das,^a^  Dipayan Bose,^a^ Somenath Banerjee,^a^ Tarun Jha,^b^ Krishna Das Saha^a*^

^a^Cancer Biology & Inflammatory Disorder Division, CSIR-Indian Institute of Chemical Biology, 4 Raja S.C. Mullick Road, Kolkata-700032, West Bengal, India

^b^Division of Medicinal and Pharmaceutical Chemistry, Department of Pharmaceutical Technology, P. O. Box 17020, Jadavpur University, Kolkata 700032, India


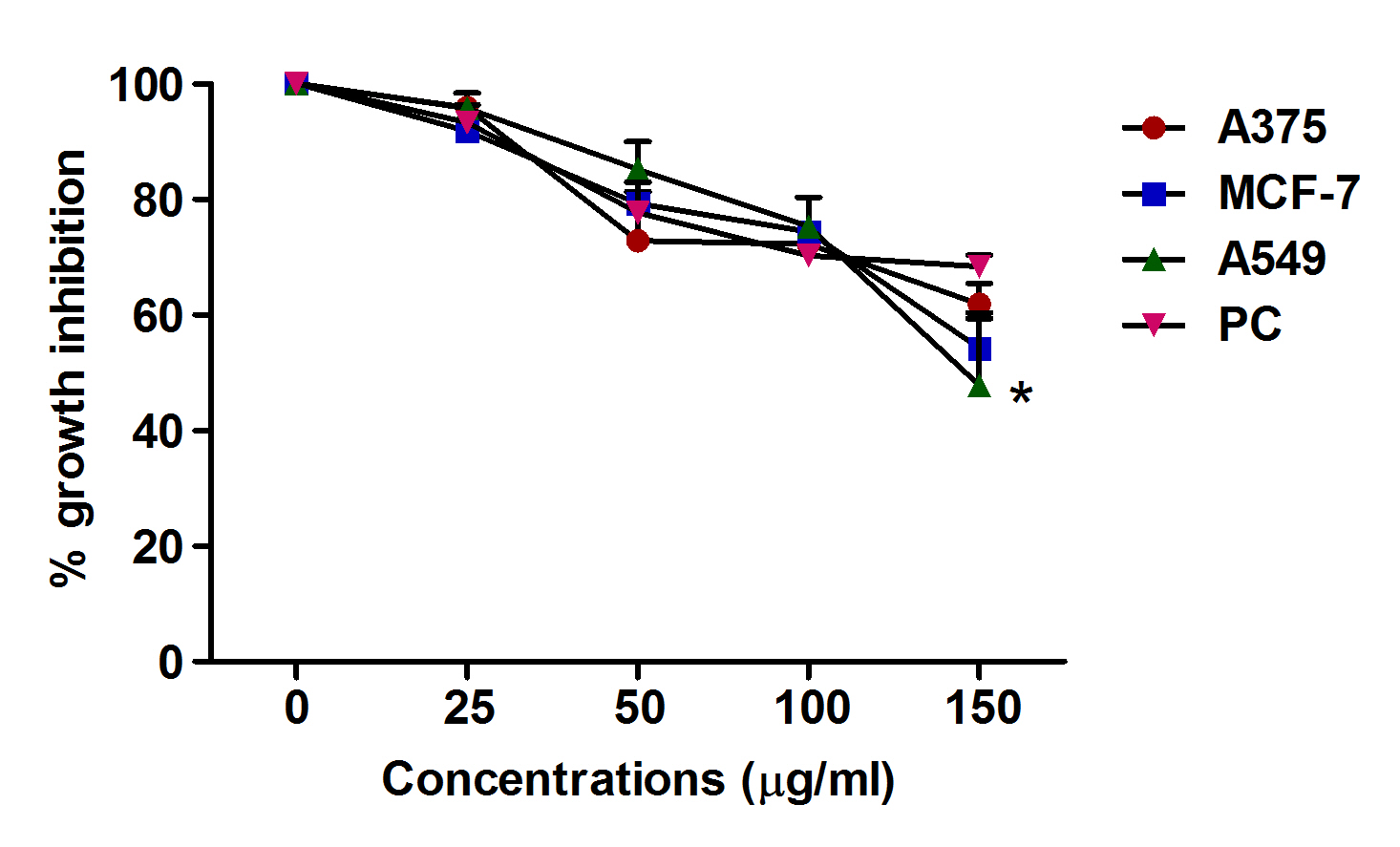

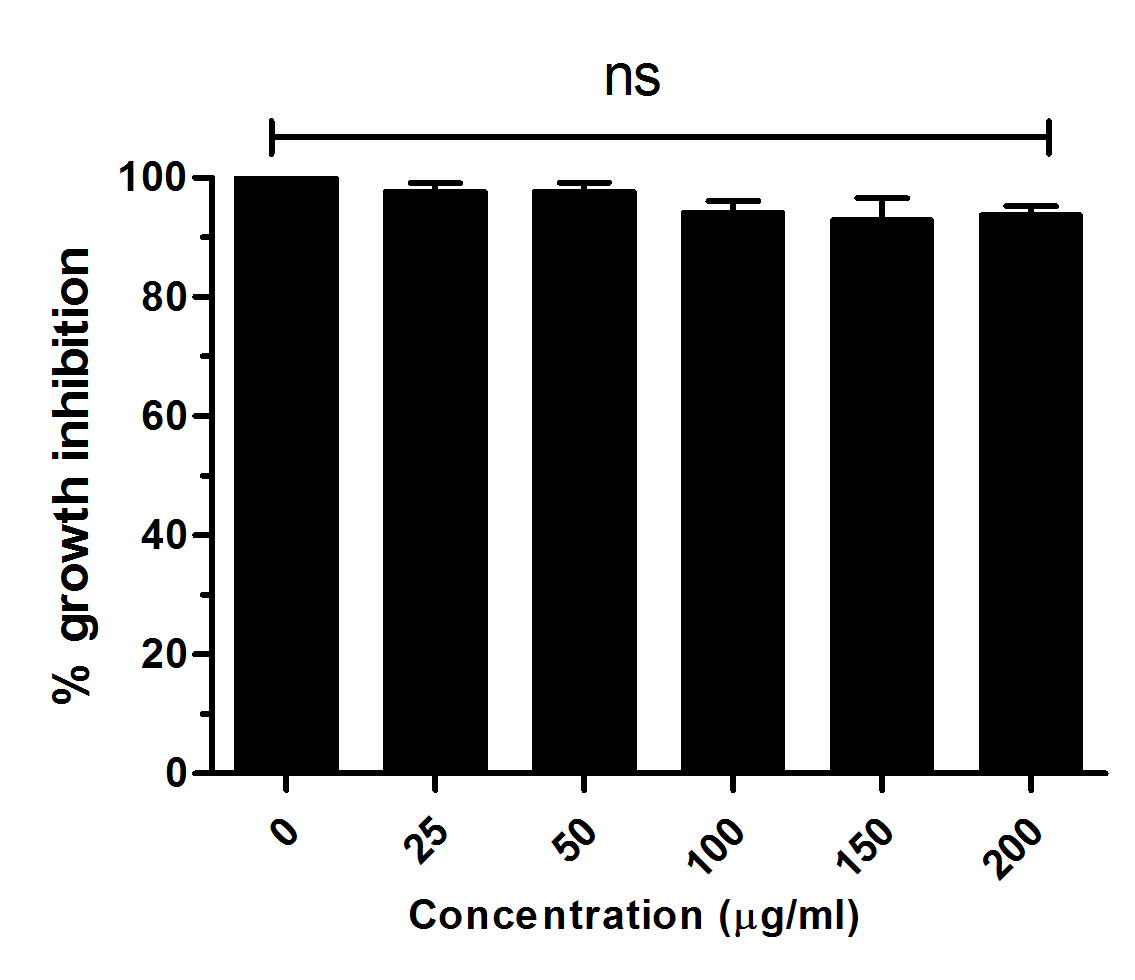


**A**

**B**

Figure S1: Growth inhibitory effect of pLLD on different cancer cells including (A) A375, MCF-7, A549, PC and (B) normal blood peripheral mononuclear cells at different concentrations (0, 25, 50, 150, 200 μg/ml) at 24 h; viability was measured by MTT assay. Data represent mean ± SEM of three experiments (** p<0.01).
