# Supplementary material for: Lipid from Infective L. donovani Regulates Acute Myeloid Cell Growth via Mitochondria Dependent MAPK Pathway
Source: PLoS One. 2015 Mar 9;10(3):e0120509. doi: 10.1371/journal.pone.0120509 (PMC4353703; doi:10.1371/journal.pone.0120509)
Supplement: S2 Fig — U937 cells were treated as indicated for measurement of intracellular GSH as described in Materials and Methods. Data represent mean ± SEM of three experiments. ** p<0.01 and *** p<0.001. (DOC) [file pone.0120509.s002.doc]

**Supplementary Information 2**

**Lipid from infective *L. donovani* regulates acute myeloid cell growth via mitochondria dependent MAPK pathway**

Nabanita Chatterjee,^a^ Subhadip Das,^a^  Dipayan Bose,^a^ Somenath Banerjee,^a^ Tarun Jha,^b^ Krishna Das Saha^a*^

^a^Cancer Biology & Inflammatory Disorder Division, CSIR-Indian Institute of Chemical Biology, 4 Raja S.C. Mullick Road, Kolkata-700032, West Bengal, India

^b^Division of Medicinal and Pharmaceutical Chemistry, Department of Pharmaceutical Technology, P. O. Box 17020, Jadavpur University, Kolkata 700032, India

Measurement of Intracellular GSH

Methods:

Intracellular GSH contents were measured using a Glutathione Assay kit (Cayman, Michigan, USA). In brief, 2×10^6^ cells were sonicated in 5% metaphosphoric acid. Particulate matters were separated by centrifugation at 10000 × 6g at 4°C. The supernatant were used for the intracellular GSH measurement as per manufacturer’s protocol.


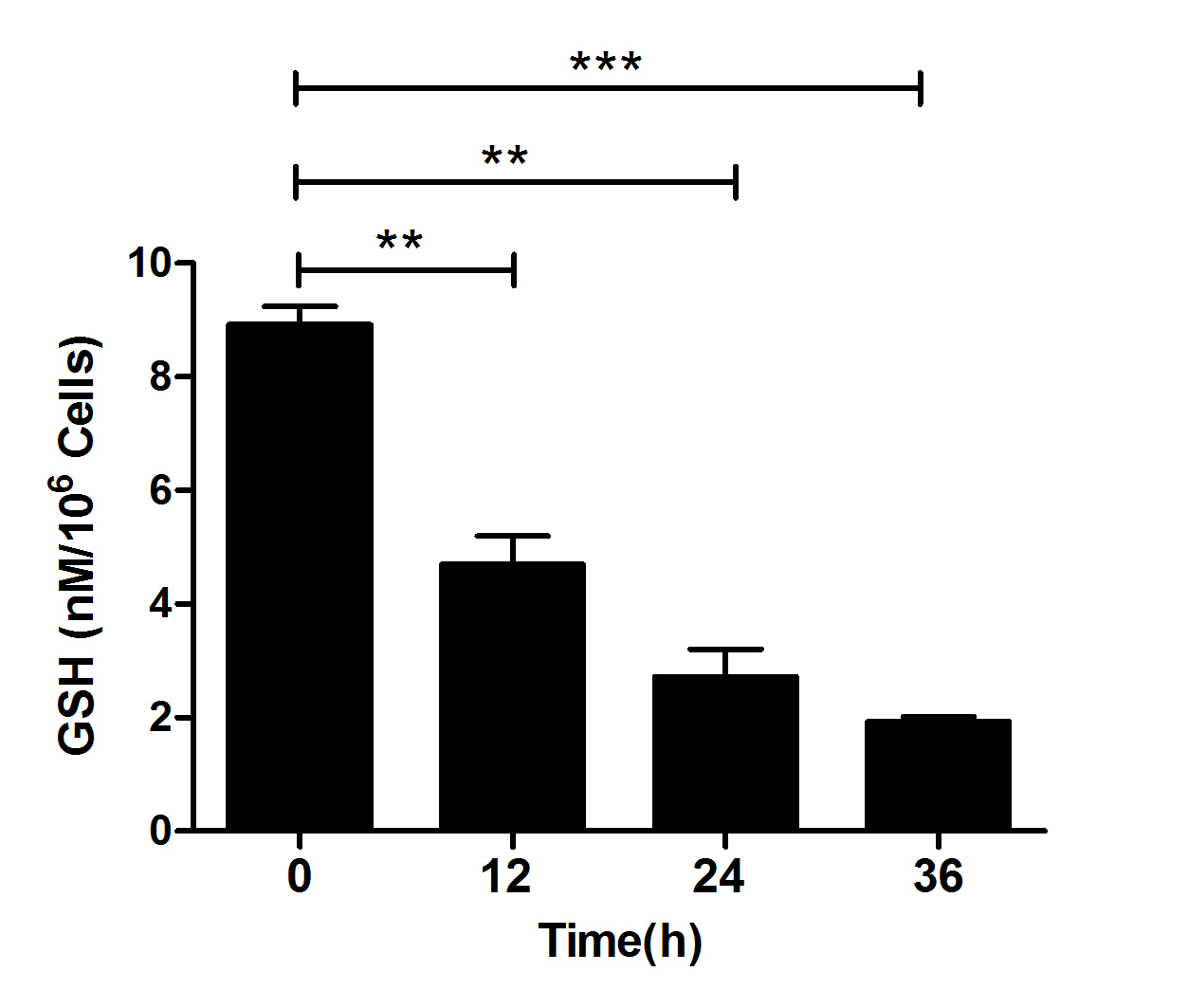


Figure S2: Effect of pLLD potentiates on intracellular GSH in U937 cells. U937 cells were treated as indicated for measurement of intracellular GSH as described in Materials and Methods. Data represent mean ± SEM of three experiments. ** p<0.01 and *** p<0.001.

Depletion of intracellular GSH might potentiate intracellular threshold of ROS and had also been exploited for cancer cell killing. Disruption of the intracellular redox state by altered GSH content affects the activation signaling pathways, which could make cancer cells susceptible to toxic insults. GSH is known to fulfill multiple defensive functions in the cell. pLLD (150 μg/ml) drastically potentiated the depletion of intracellular GSH in U937 cells leading to generation of ROS in time dependent manner as found in Figure S2.
