# Supplementary material for: Lipid from Infective L. donovani Regulates Acute Myeloid Cell Growth via Mitochondria Dependent MAPK Pathway
Source: PLoS One. 2015 Mar 9;10(3):e0120509. doi: 10.1371/journal.pone.0120509 (PMC4353703; doi:10.1371/journal.pone.0120509)
Supplement: S3 Fig — The data are reported as the mean ± SEM of triplicate experiments (*P>0.05; ** P>0.01). (B) Assessment of MAPK pathway with inhibitors PD098059, SB203580 and SP600125 by pLLD was done in a time dependent manner by western blot analysis. The data are reported as the mean ± SEM of triplicate experiments (*P>0.05; ** P>0.01; *** P>0.001). (DOC) [file pone.0120509.s003.doc]

**Supplementary Information 3**

**Lipid from infective *L. donovani* regulates acute myeloid cell growth via mitochondria dependent MAPK pathway**

Nabanita Chatterjee,^a^ Subhadip Das,^a^  Dipayan Bose,^a^ Somenath Banerjee,^a^ Tarun Jha,^b^ Krishna Das Saha^a*^

^a^Cancer Biology & Inflammatory Disorder Division, CSIR-Indian Institute of Chemical Biology, 4 Raja S.C. Mullick Road, Kolkata-700032, West Bengal, India

^b^Division of Medicinal and Pharmaceutical Chemistry, Department of Pharmaceutical Technology, P. O. Box 17020, Jadavpur University, Kolkata 700032, India

**A**


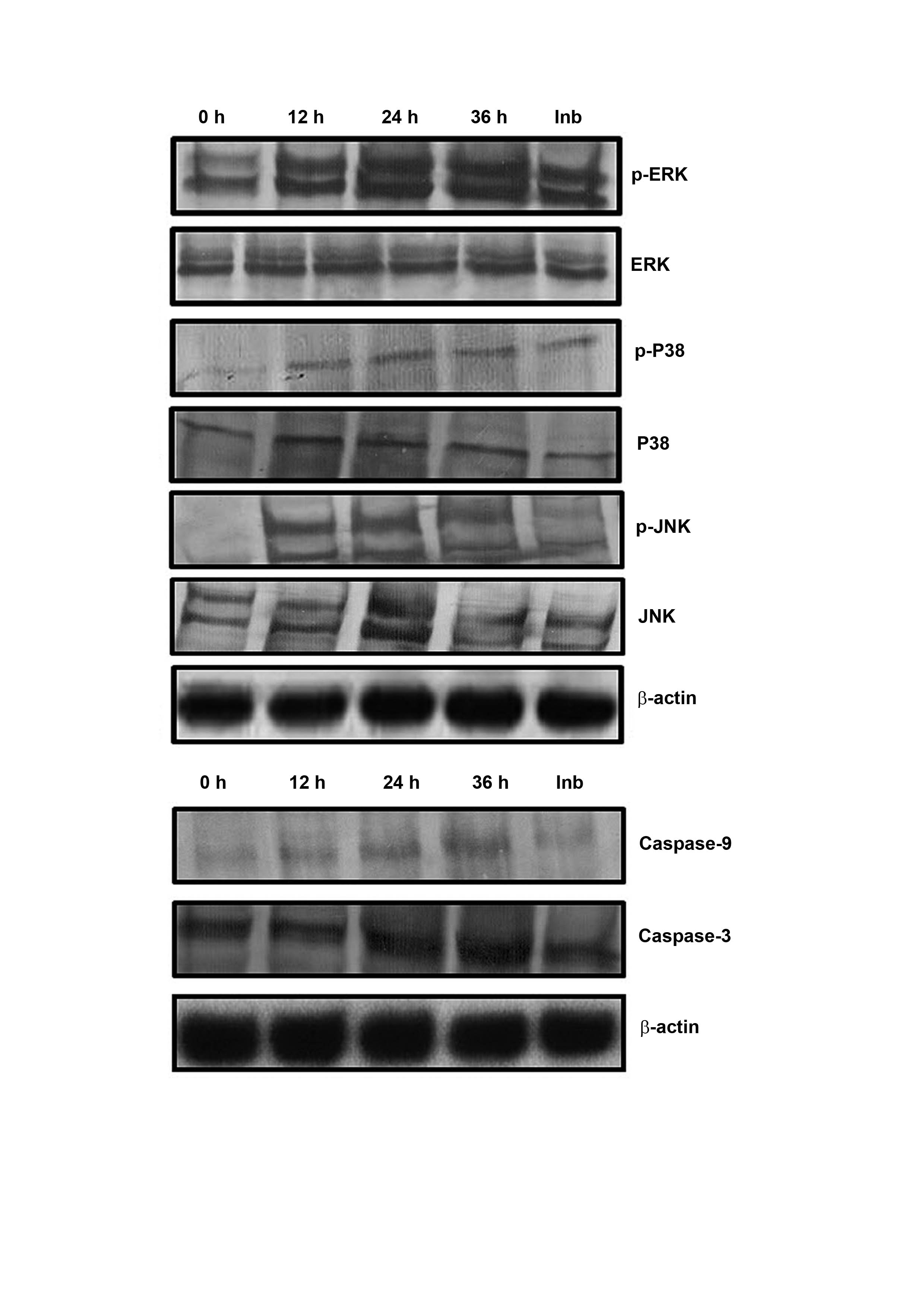

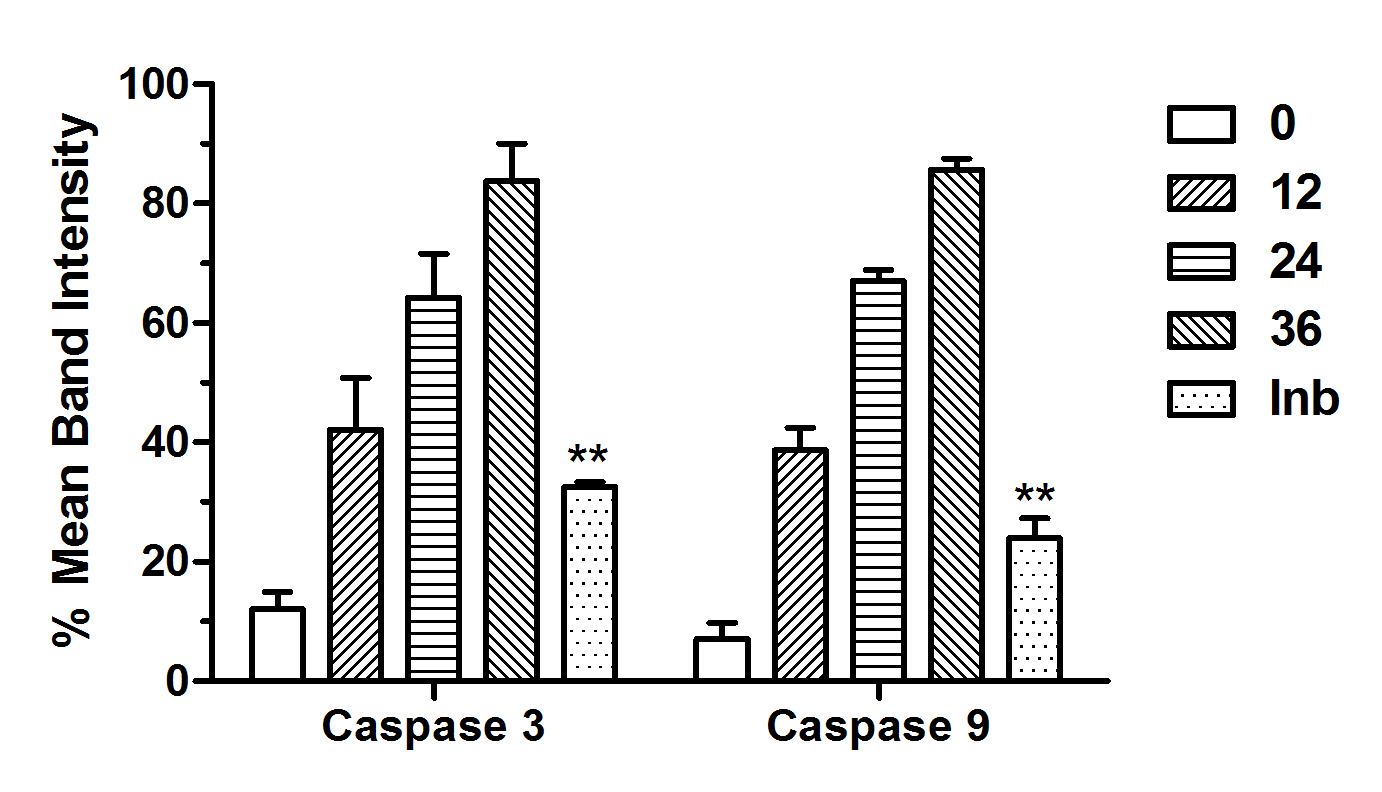


Figure S3: (A) Assessment of caspase- 9 and 3 activation with inhibitors Z-DEVD-FMK, Z-LEDH-FMK by pLLD was done in a time dependent manner by western blot analysis. The data are reported as the mean ± SEM of triplicate experiments (*P>0.05; ** P>0.01).


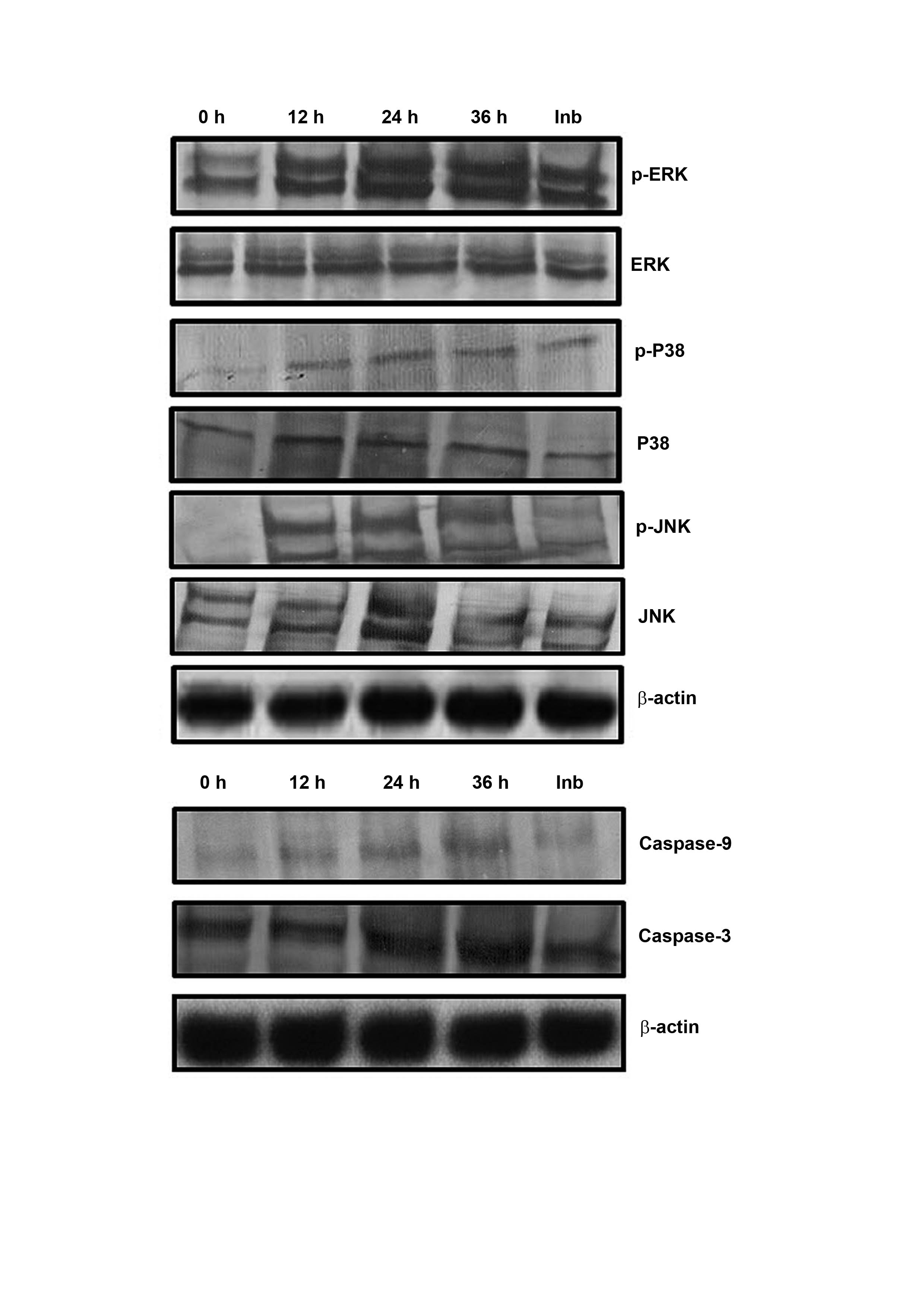

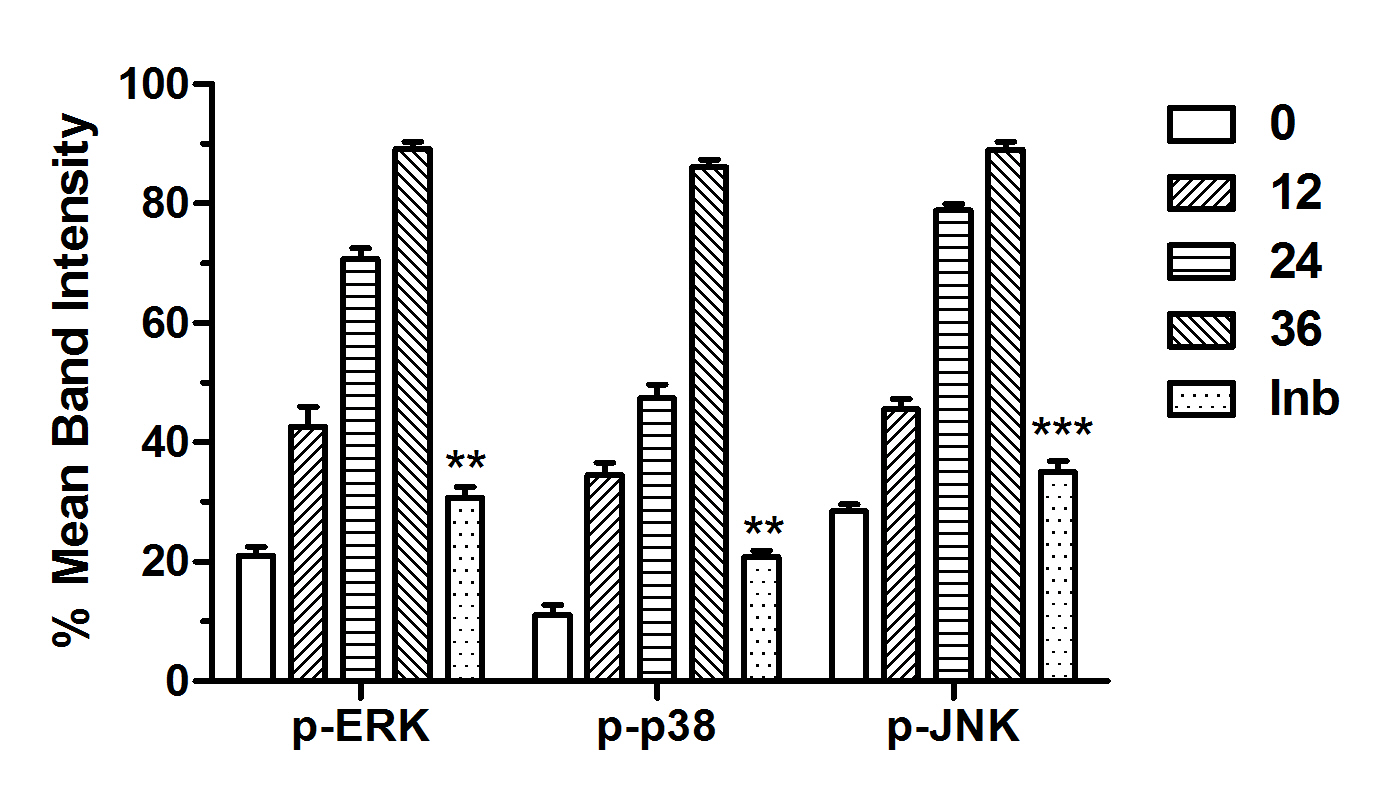


**B**

Figure S3: (B) Assessment of MAPK pathway with inhibitors PD098059, SB203580 and SP600125 by pLLD was done in a time dependent manner by western blot analysis. The data are reported as the mean ± SEM of triplicate experiments (*P>0.05; ** P>0.01; *** P>0.001).
